# Improvement of Executive Function after Short-Term Administration of an Antioxidants Mix Containing Bacopa, Lycopene, Astaxanthin and Vitamin B12: The BLAtwelve Study

**DOI:** 10.3390/nu13010056

**Published:** 2020-12-27

**Authors:** Francesca Crosta, Amanda Stefani, Francesco Melani, Paolo Fabrizzi, Andrea Nizzardo, Davide Grassi, Raffaella Bocale, Stefano Necozione, Francesca Lombardi, Vanessa Castelli, Arrigo F. G. Cicero, Annamaria Cimini, Claudio Ferri, Giovambattista Desideri

**Affiliations:** 1 Department of Health, Life and Environmental Sciences, University of L’Aquila, 67100 L’Aquila, Italy; francydoc@gmail.com (F.C.); stefaama@icloud.com (A.S.); davide.grassi@cc.univaq.it (D.G.); stefano.necozione@cc.univaq.it (S.N.); francesca.lombardi@univaq.it (F.L.); vanessa.castelli@univaq.it (V.C.); annamaria.cimini@univaq.it (A.C.); claudio.ferri@cc.univaq.it (C.F.); 2 Clinical Research, Menarini Group, 50131 Florence, Italy; fmelani@menarini.it (F.M.); pfabrizzi@menarini.it (P.F.); anizzardo@menarini-ricerche.it (A.N.); 3 Division of Endocrine Surgery, “Agostino Gemelli” School of Medicine, University Foundation Polyclinic, Catholic University of the Sacred Heart, 00198 Rome, Italy; raffaella.bocale@policlinicogemelli.it; 4 Hypertension and Cardiovascular Risk Factors Research Group, Medical and Surgical Sciences Department, Sant’Orsola-Malpighi University Hospital, 40138 Bologna, Italy; arrigo.cicero@unibo.it

**Keywords:** antioxidants, oxidative stress, cognitive function, bacopa, lycopene, astaxanthin, vitamin B12

## Abstract

During the last few years increasing interest has been focused on antioxidants as potentially useful agents in the prevention of the onset and progression of cognitive dysfunction. In this randomized, double-blind, controlled, parallel arm study, the effects of daily consumption of an antioxidant mix on cognitive function in healthy older adults were evaluated. After a 1 week run-in period, 80 subjects aged 60 years or more, and with no evidence of cognitive dysfunction, were randomly allocated to a mix of four bioactive compounds (bacopa, lycopene, astaxanthin, and vitamin B12) or matched placebo, taken orally once a day for 8 weeks. The primary objective of the study was to evaluate the changes in trial making test (TMT) scores from baseline to 8 weeks of treatment, analyzed in the following hierarchical order: TMT-B, TMT-A, and TMT-B minus TMT-A. TMT-B increased in the control group (+3.46 s) and decreased in the active group (−17.63 s). The treatment difference was −21.01 s in favor of the active group (95% C.I. −26.80 to −15.2, *p* < 0.0001). The decrease in TMT-A was significantly higher in the active group (−6.86 s) than in the control group (−0.37 s). TMT-B minus TMT-A increased in the control group (+3.84 s) and decreased in the active group (−10.46 s). The increase in letter fluency in the verbal fluency test (VFT) was also significantly higher in the active group and statistically significant (+5.28 vs. +1.07 words; *p* < 0.001). Our findings provide encouraging evidence that regular dietary supplementation with bacopa, lycopene, astaxanthin, and vitamin B12 may be an effective dietary approach for counteracting cognitive changes associated with brain aging.

## 1. Introduction

Long-term oxidative stress is believed one of the most important factors contributing to the decline of cognitive function often observable with aging [1]. Oxidative stress, due to the generation of free radicals resulting from normal metabolism, is usually maintained at a low level by the antioxidant system. However, in some conditions the oxidant/antioxidant balance can be perturbed by increased generation of reactive oxygen species and/or decreased endogenous ability to counteract them [1]. Brain tissue is highly sensitive to oxidative stress because it has a high request for oxygen and has a relative weakness of antioxidant systems. Furthermore, the brain also contains high levels of polyunsaturated fatty acid, making it more vulnerable to oxidative injuries [1]. Altered mitochondrial function, the amyloid β peptides and the presence of unbound trace metal ions represent the most investigated potential sources of oxidative stress in the brain [2,3]. Depending on the biomolecules attacked by reactive oxygen species, oxidative stress can promote peroxidation of protein, lipids, and nucleic acids thus favoring the onset and progression of cognitive dysfunction during aging [1].

During the last few years increasing interest has been focused on bioactive compounds exhibiting antioxidant and anti-inflammatory functionality, including flavonoids and carotenoids, such that their intake may have protective effects against the onset and progression of cognitive dysfunction by maintaining and restoring oxidative and inflammatory balance [4,5,6].

Epidemiologic studies suggest that a regular intake of flavonoids could be associated with better cognitive function [7], and better dose-dependent cognitive performance in normal aging [8,9]. Randomized controlled trials also demonstrated that daily consumption of flavanol-rich cocoa drink positively affects cognition, leading to improvements in cognitive performance both in older adults with early memory decline [10], and in cognitively intact elderly subjects [11]. Furthermore, the beneficial effects of polyphenolic compounds have been attributed to their ability to exert antioxidant actions [12], and also to a range of actions involving the ability to protect vulnerable neurons, enhance neuronal function, and improve vascular health and metabolic profile [13].

Population-based and cross-sectional studies have shown that higher intake of carotenoids is associated with better cognitive performance in healthy individuals [14,15,16]. Other smaller studies have also observed positive associations between the xanthophyll carotenoids and cognitive domains including memory [17], executive function [15], and language [18]. Recent interventional studies in healthy individuals have observed improvements in various domains of cognition following carotenoid supplementation. These include episodic memory [19], attention [20], and processing speed [21].

According to the above evidence, a recent meta-analysis of nine studies demonstrated that increased consumption of fruit and vegetables was associated with significant reduction in the risk of cognitive impairment and dementia [22]. Moreover, antioxidant and anti-inflammatory dietary patterns, such as the Mediterranean diet, and dietary approaches to stop hypertension (DASH) have been associated with better cognitive function [23].

Fruit and vegetables contain numerous bioactive compounds exhibiting antioxidant and anti-inflammatory functionality, which can cooperate in improving cognitive function. Due to the different biological effects of nutrients in foods, it is conceivable that a mixture of bioactive compounds known for inhibiting free radical formation and/or favorably influencing brain function might be more effective than individual antioxidants. Indeed, there is some evidence of biological synergism between some carotenoids and other antioxidants, with a positive effect on the improvement of cognitive function of elderly people [24,25].

Starting from this knowledge, we have designed an in vitro/in vivo research program aiming to evaluate whether a food supplement containing bacopa, lycopene, astaxanthin, and vitamin B12 could be effective in improving brain health/functioning. Lycopene, a lipid-soluble carotenoid compound, well represented in tomatoes and red fruits, including watermelon, pink grapefruit, and guava, has been proposed as a brain protective agent [26]. In vitro studies indicated that lycopene protects against neuronal death induced by different neurotoxic compounds, including 1-methyl-4-phenylpyridinium (MPP+), methylmercury, amyloid β, trimethyltin, and 6-hydroxydopamine [27,28]. Furthermore, animal experiments using rat models demonstrated that lycopene prevents brain injury caused by focal or global ischemia and reperfusion [29], and alleviates cognitive dysfunction induced by colchicine and rotenone [30]. A population-based follow-up study demonstrated that males in the highest quartile of serum lycopene concentrations exhibited 59% and 55% lower risks of ischemic stroke when compared with males in the lowest quartile [31]. Astaxanthin is a xanthophyll carotenoid nutrient known for having potent antioxidant and anti-inflammatory actions due to its molecular properties that precisely position it within cell membranes and circulating lipoproteins [32,33]. Astaxanthin has shown a variety of brain benefits under experimental conditions [32,33]. *Bacopa monnieri* is a creeping herb extensively investigated for its pharmacological and therapeutic effects. Its ethanol extract contains a mixture of triterpenoid saponins, designated as bacosides A and B [34,35,36]. In vitro studies using *Bacopa monnieri* have shown that it inhibits free radical formation and deoxyribonucleic acid damage in a dose dependent manner [37]. Promising indications for use in humans include improving cognition in the elderly and in subjects with neurodegenerative disorders [38]. Notably, it has been reported that in a short-term randomized, placebo-controlled clinical trial (6 weeks in adults without cognitive impairment) there was a significant improvement in tests relating to the cognitive performance upon *Bacopa monnieri treatment* [39]. Furthermore, *Bacopa monnieri* was able to improve some aspects of cognitive functions in a 6-month trial in Alzheimer’s disease patients [40]. In particular, different components of the mini-mental state examination scale, including orientation of place, person and time, attention, and their language ability, at the end of trial resulted as significantly improved. Vitamin B12, also called cobalamin, is a water-soluble vitamin that has a key role in the normal functioning of the brain and nervous system, and for the formation of red blood cells [41]. Serum levels in the subclinical low-normal range (<250 pmol/L) are associated with Alzheimer’s disease, vascular dementia, and Parkinson’s disease, while some evidence suggests that vitamin B12 administration might be useful in preserving brain health [42]. A randomized, placebo-controlled trial in women of different ages, demonstrated that the short-term supplementation of vitamin B12 had a significant positive effect on some measures of memory performance [43]. Interestingly, for a combination of astaxanthin with β-carotene, or astaxanthin with lycopene, significant antioxidant synergism was observed for initiation of oxidation in the lipid phase [44,45]. Moreover, the cognitive effects of a short-term dietary supplement composed by the combination of *Bacopa monnieri*, astaxanthin, phosphatidylserine, and vitamin E in subjects with mild cognitive impairment revealed significant improvement in memory and cognitive tests [46]. In line with these results, our in vitro studies demonstrated a synergistic effect combining these four bioactive compounds. Interestingly, the combination of these four compounds was able to counteract the deleterious effect of hydrogen peroxide in human neuronal differentiated cells, by increasing cell viability and the proteins involved in neuroprotective pathways, and restoring proteins involved in cell death pathways [47]. Based on these encouraging results, the purpose of the current study was to evaluate if the same mix of bioactive compounds, orally administered for 8 consecutive weeks, can favorably influence cognitive performance in a target population with no evidence of cognitive dysfunctions.

## 2. Materials and Methods

### 2.1. Participants

Eighty subjects, aged 60 years or more, were randomly extracted among those included in a list of 160 potentially recruitable subjects provided by general practitioners of our district on the basis of the following exclusion criteria: clinically significant co-existing medical conditions (cardiovascular disease, cerebrovascular events, overt dementia defined by mini mental state examination (MMSE) <27, or other neurological disorders, thyroid disorders, or inflammatory diseases); geriatric depression scale (GDS) >11, in order to avoid confounding due to the influence of concomitant depression on the performance in cognitive tests; current smokers; habitual users of antioxidant supplements (including vitamins C and E); habitual consumers of chocolate or other cocoa products (daily consumption of any amount); treatment with medications known to have antioxidant properties (including statins and glitazones) or to interfere with cognitive functions (including benzodiazepines and antidepressants); history of hypersensitivity to any component of the study medications. Individuals who were participating in or had participated in another clinical trial within the previous three months were also excluded. All participants were requested to be able to provide written informed consent to the study. Twelve subjects refused to participate.

### 2.2. Study Design

To investigate the impact of regular consumption of a new food supplement composed of four bioactive ingredients on cognitive function in non-demented elderly individuals, an 8-week, double-blind, randomized, controlled, parallel arm study was conducted. All participants met with a dietician 1 week before the randomization in order to evaluate current diet habits, and correct any nutritional insufficiencies, so that their diet was relatively standardized. All participants were encouraged to continue with their usual physical activity throughout the study period. After a 1-week run-in period, participants were randomly allocated to a mix of the four bioactive compounds (bacopa, lycopene, astaxanthin, and vitamin B12) or matched placebo, taken orally once a day for 8 weeks (a detailed description of the food supplement composition and its placebo can be found in Appendix A, Appendix A.1). This time period was considered adequate to detect eventual effects of the antioxidant mix on executive function on the basis of previous experiences from our group [10,11]. All the participants were invited to bring back unused tablets and empty boxes to check compliance. This latter was calculated as the percentage of the number of tablets actually taken by the subject over the number of tablets expected to have been taken.

The study has been registered at ClinicalTrials.gov ID:NCT03825042.

### 2.3. Study Outcomes

The primary objective of the study was the change in trial making test (TMT) scores from baseline to 8 weeks of treatment, analyzed in the following hierarchical order: TMT-B, TMT-A, and TMT-B minus TMT-A.

Secondary objectives were changes from baseline to 8 weeks of treatment in verbal fluency test (VFT) score, Montreal cognitive assessment (MoCA) score, MMSE score, and Rey auditory verbal learning test (AVLT) score.

Changes of metabolic parameters, including glucose, insulin, homeostatic model assessment for insulin resistance (HOMA-IR), total cholesterol, low density lipoprotein (LDL) cholesterol, high density lipoprotein (HDL) cholesterol, triglycerides and uric acid, and plasma markers of oxidative stress, including 8-iso-Prostaglandin F2alpha and plasma malondialdehyde (MDA) from baseline to week 4 and 8 were also evaluated as secondary objectives.

### 2.4. Cognitive Function Assessment

Cognitive testing was performed at baseline and after 8 weeks (±2 days) using well-validated standardized tests: TMT, VFT, MMSE, MoCa, and AVLT (details on neuropsychological tests used in this study can be found in Appendix A, Appendix A.2).

### 2.5. Blood Pressure Measurement

Before neuropsychological testing, clinical systolic and diastolic blood pressures were recorded in the morning with the use of a validated oscillometric device with appropriately sized cuffs (Omron 705 CP; Omron Matsusaka) on the nondominant upper arm. These evaluations were performed by staff blinded to the study protocol. At each visit, participants rested 15 min in a seated position, the first blood pressure measurement was taken but discarded, and the subsequent three consecutive blood pressure readings, taken at 3-min intervals, were recorded. The average of these latter measures was considered for statistical analysis.

### 2.6. Laboratory Analysis

Within 24 h of neuropsychological testing, blood samples were drawn from each participant after an overnight fasting period for determinations of lipid profile and fasting plasma glucose and insulin. The HOMA-IR index (fasting serum insulin (mU/L) × fasting plasma glucose (mmol/L)/22.5) was calculated from fasting glucose and insulin concentrations as a marker of insulin resistance. The plasma levels of 8-isoprostanes and malondialdehyde were measured by using an 8 Isoprostane ELISA kit (ab175819, Abcam Cambridge, United Kingdom) and an MDA assay kit, competitive ELISA (ab238537, Abcam), respectively, according to the manufacturer’s instructions. The absorbance was measured by spectrophotometric reading at 450 nm using a microplate reader (Bio-Rad Hercules, CA, USA).

### 2.7. Statistical Analysis

The primary objective of this study was assessed using three primary variables (changes in TMT-B, TMT-A, and TMT-B minus TMT-A) in a hierarchical order in the intent-to-treat population (ITT) defined as all randomized subjects receiving at least a treatment dose and having the post-randomization efficacy evaluation. According to the “points to consider on multiplicity issues in clinical trials”, issued by the Committee for Proprietary Medicinal Products (CPMP-2002), no adjustment for multiplicity was needed, since a hierarchical approach was adopted. The study was based on an estimated sample size of 68 subjects, with a ratio of 1:1 for the 2 treatment groups, which was calculated to be adequate to achieve a 90% power to detect a large effect size (f = 0.40) using an ANCOVA model with the treatment group (main effect) and baseline as covariates, and an α of 0.05 between treatment and control (G*power version 3.1.9.2). To allow for a possible 15% dropout rate, 80 subjects were enrolled. Once eligibility was established, subjects were randomized to receive either a mix of the four bioactive compounds or placebo, in a 1:1 ratio and a double-blind manner. The primary endpoint and all the other continuous variables were descriptively summarized by the number of subjects (N), mean, standard deviation, median, minimum, maximum; all the other continuous variables were analyzed using an ANCOVA model with the treatment group (main effect) and baseline as covariates. As an additional covariate for testing the homogeneity of the regression coefficients, the interaction term of treatment*baseline was included in the model and removed if not significant. Categorical variables were summarized by the number (N) and the proportion of subjects (%), and tested by using a chi-square test or the Fisher exact test if necessary. The significance level of statistical tests was set at 0.05. Missing values were not imputed. No interim analysis was planned. The statistical analysis was performed using SAS 9.4 for Windows (SAS Institute Inc., Cary, NC, USA).

## 3. Results

### 3.1. General Characteristics of the Study Population 

General characteristics of the study population are shown in Table 1. According to the selection criteria none of participants had overt cognitive dysfunction or depression. All the assessed variables at baseline were similar in the two study groups, indicating an adequate randomization procedure.

### 3.2. Primary Endpoints 

**Figure 1:**
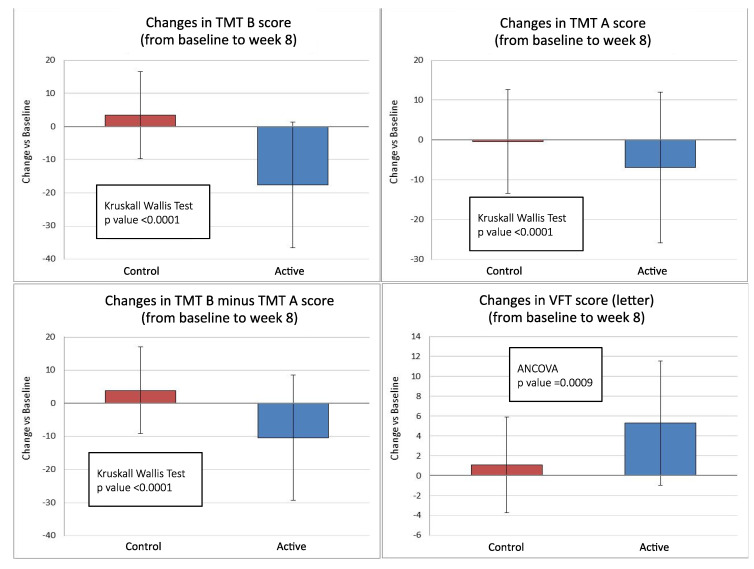
Changes in trail making test (TMT) A, TMT-B, and TMT-B minus TMT-A, and verbal fluency test (VFT) score from baseline to 8 weeks of treatment in active (antioxidants mix, *n* = 38) and control (placebo, *n* = 40) groups (ITT population).

The differences between treatment groups in changes between baseline and week 8 of the three primary endpoints were statistically significant (*p* < 0.001) in favor of the active group. TMT-B increased in the control group (+3.46 s) and decreased in the active group (−17.63 s) (Table 2). The treatment difference was −21.01 s in favor of the active group (95% C.I. −26.80 to −15.2, *p* < 0.0001) (Figure 1). The decrease in TMT-A was significantly higher in the active group (−6.86 s) than in the control group (−0.37 s) (Table 2). The treatment difference was −6.08 s in favor of the active group (95% C.I. −8.54 to −3.61, *p* < 0.0001) (Figure 1). TMT-B minus TMT-A increased in the control group (+3.84 s) and decreased in the active group (−10.46 s) (Table 2). The treatment difference was −14.56 s in favor of the active group (95% C.I. −20.02 to −9.11, *p* < 0.0001) (Figure 1).

### 3.3. Secondary Endpoints

The increase in letter fluency of VFT was significantly higher in the active group and statistically significant (+5.28 vs. +1.07 words; *p* < 0.001) (Table 2). The treatment difference was +4.33 correct words in favor of the active group (95% C.I. +1.83/+6.82, *p* = 0.0009) (Figure 1). No other statistically significant differences were detected in the other neuropsychological tests performed (Table 2).

**Figure 2:**
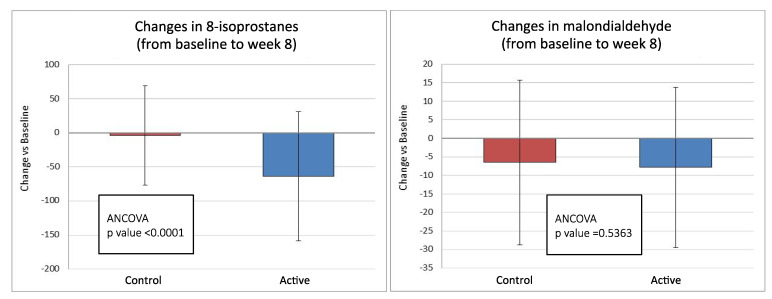
Changes in circulating levels of 8-isoprostanes and malondialdehyde from baseline to 8 weeks of treatment in active (antioxidants mix, *n* = 38) and control (placebo, *n* = 40) groups (ITT population).

A stunning and statistically significant difference between treatment groups was observed in the changes of 8-isoprostane levels between baseline and both week 4 and week 8. The decrease in the control group was −9.82 and −4.14 pg/mL at week 4 and week 8, respectively, and −57.08 and −63.65 pg/mL in the active group (*p* < 0.001). The treatment difference was −65.31 pg/mL in favor of the active group (95% C.I. −91.72 to −38.8, *p* < 0.0001) (Figure 2).

A statistically significant difference in the changes in plasma malondialdehyde levels between baseline and week 4 was observed: mean plasma malondialdehyde level was decreased by 5.22 and 10.91 pmol/mL in the control and active group, respectively (*p* < 0.05). This difference was no longer evident at week 8 (−2.67 pmol/mL in favor of the active group, 95% C.I. −11.22/−5.89, *p* = 0.5363) (Figure 2).

A significant difference between treatment groups was observed for changes of insulin blood level from baseline to 8 weeks: −2.31 vs. +0.70 in the placebo and active group, respectively; *p* < 0.05 (Table 3). Notably, this significance was likely driven by a huge reduction in plasma insulin levels (−25.3 UI/L) in a single patient under placebo. The same behavior was observed for HOMA-IR, but the difference was not statistically significant.

No other significant differences in the changes of metabolic parameters between baseline and following visits were observed (Table 3).

### 3.4. Safety Results 

Only two adverse events were observed during the study, both in the active group. The first subject experienced an exacerbation of sinusitis. The event was not serious, not related to the study treatment, no action on the study treatment was taken, and it resolved in 2 days. The second subject experienced an acute virus, hepatitis E. The event was serious but clearly not related to the study treatment. The study treatment was permanently discontinued, and the virus resolved in 8 days.

### 3.5. Adherence to the Study Protocol

Two subjects withdrew prematurely, both in the active treatment group: one subject was not able to attend scheduled visits after the randomization and then withdrew consent, but agreed to perform the end of study visit; one subject experienced an acute virus, hepatitis E, and permanently discontinued the study treatment after 4 weeks from randomization.

The treatment compliance was 100% for nearly all the subjects, exceptions being one subject who did not bring back unused tablets nor empty boxes at week 4, and one subject who forgot to take one tablet.

## 4. Discussion

This study provides encouraging evidence that the daily consumption of an antioxidant mix, containing bacopa, lycopene, astaxanthin, and vitamin B12 for eight weeks could favorably influence cognitive function in healthy older adults. Indeed, we found that cognitive performance was improved with regular antioxidant mix consumption, without evidence of any relevant adverse effects. This is the first well-controlled study of its kind in cognitively-intact elderly adults to demonstrate such enhancements. In addition to these cognitive improvements, the regular dietary inclusion of the antioxidant mix led to significant reductions in some oxidative stress markers.

Long-term oxidative stress appears to be a major factor in declining cognition [1,48]. It may be modifiable through diet and/or antioxidant supplements [49]. Antioxidant use as a potential intervention strategy for the prevention and/or treatment of cognitive decline and dementia has been of interest for many years [10,11,50,51,52]. Nonetheless, epidemiologic data on antioxidant vitamins and cognition are not consistent. Some large observational investigations have reported that high intake or high plasma levels of various antioxidants were associated with better cognitive performance [25,53,54,55,56,57,58] and a reduced risk of Alzheimer’s disease and all-cause dementia [59,60,61,62], but several randomized trials have not found neuroprotection [63,64,65,66,67]. In the Prevention of Alzheimer’s Disease by Vitamin E and Selenium trial (PREADViSE) neither vitamin E nor selenium had a significant preventive effect on the incidence of dementia [68]. Similarly, the Physicians’ Health Study II did not demonstrate an impact of short-term beta carotene supplementation on cognitive performance [69].

The current study sheds new light on this relevant topic, providing interesting evidence of an improvement of some aspects of neurocognitive performance in response to the regular consumption of an antioxidant mix in healthy older adults. The benefits included improvements in text processing speed, executive function, and working memory, as indicated by the improvement in the performance in TMT A, TMT B, TMT B minus TMT A, and VFT scores; a battery of neuropsychological tests commonly used as a measure of executive function. In this regard, age-related cognitive declines are generally understood in terms of a range of mechanisms, including processing speed, working memory, inhibition, and cognitive control that can all be categorized as executive processes [70,71]. These results could suggest a particular sensitivity of this subset of neuro-psychological functions to the benefits of antioxidants. Thus, our study provides the first interesting suggestion that dietary inclusion of a mix containing bacopa, lycopene, astaxanthin, and vitamin B12 could be effective in ameliorating age-related cognitive declines.

It is unclear from the current study how the daily consumption of the tested antioxidants mix might contribute to the observed cognitive effects. Some previously published studies have found that antioxidant intake can lead to a significant improvement in nitric oxide-dependent endothelial function within the peripheral vasculature [52]. Thus, one possibility is that the improvements in cognitive performance observed in the current study are a direct consequence of a favorable influence of the antioxidant mix on endothelial function, which led to changes in cerebrovascular blood flow [52]. In addition, direct neuroprotective effects of the tested antioxidant mix can be also considered. Indeed, evidence from our in vitro study supports that the compounds included in the tested antioxidant mix were able to protect human neuronal differentiated cells against hydrogen peroxide-induced injury, counteracting its cytotoxic effects [47]. Moreover, the antioxidant mix upregulated the expression of the neurotrophic pathways, while decreasing death pathways [47]. This implies that this treatment was able to restore in neuronal-like cells the correct oxidative balance, by upregulating reactive oxygen species scavenger enzymes and, probably as a consequence, reducing cell death execution and improving cell survival. It is worth noting that in neurodegeneration, an impairment of brain-derived neurotrophic factor (BDNF) occurs, paralleled by cognitive decline and decrease of synaptic plasticity and memory loss. In this context, is important to underline that the antioxidant mix was able to increase BDNF in vitro, as well as proteins involved in synaptic plasticity and in the maintenance of synaptic morphology [47].

The last interesting finding of our study was the significant reduction of circulating levels of plasma 8-isoprostane levels after antioxidant mix consumption. These compounds are generated from arachidonic acid through a process of non-enzymatic free radical-catalyzed lipid peroxidation, thus representing an established marker of oxidative stress [72]. Furthermore, lipid peroxidation products are able to promote several vascular responses, including sustained vasoconstriction [72], platelet activation [73], and vascular inflammation [74], which could play a role in reducing brain tissue perfusion. Increased concentrations of isoprostanes have been demonstrated in plasma, urine, and cerebrospinal fluid from patients with Alzheimer’s disease and, to a lesser extent, in subjects with mild cognitive impairment, suggesting a parallelism between the degree of oxidant/antioxidant unbalance and the severity of cognitive dysfunction [75]. In this regard, Galasko et al. [76] demonstrated a significant reduction of 8-isoprostanes levels in cerebrospinal fluids after 16 weeks of antioxidant administration in subjects with probable Alzheimer’s disease. In addition, reduction of plasma 8-isoprostanes level has been demonstrated to contribute to the improvement of cognitive function in patients with mild cognitive impairment after 8 weeks of cocoa flavanol supplementation [10]. Thus, the relevant reduction of circulating levels of 8-isoprostanes could have contributed to the amelioration of cognitive performance observed after the antioxidant mix supplementation.

The potential clinical relevance of our results requires some consideration. First, we cannot completely exclude the possibility that other dietary components could have influenced to some extent our findings, since we did not perform a dietary assessment. However, all participants had been instructed to maintain their usual lifestyle and intake of fruits and vegetables, and to avoid any food supplements. Thus, we are confident that this potential confounder should not be relevant in the interpretation of our data. In addition, due to the study design, it is difficult to determine whether or not there was synergy among the four tested components of the antioxidant mix, or if the effects were due to one of more of these bioactive compounds. However, the exploratory in vitro study clearly demonstrated a synergistic effect of the four bioactive compounds in protecting human neuronal differentiated cells from H_2_O_2_ cytotoxic effects, by increasing cell viability and proteins involved in neuroprotective pathways, and restoring proteins involved in cell death pathways [47]. Although in vitro findings cannot be directly translated in vivo, the existence of a synergistic effect in vivo seems to be very plausible.

## 5. Conclusions

The results of the current study indicate that the regular intake of an antioxidant mix containing bacopa, lycopene, astaxanthin, and vitamin B12 can improve aspects of cognitive performance among healthy elderly subjects. These findings provide encouraging evidence that regular dietary supplementation with bacopa, lycopene, astaxanthin, and vitamin B12 may be an effective dietary approach for counteracting cognitive changes associated with brain aging.

## Figures and Tables

**Table 1:** General characteristics of the study population.

	Placebo (*n* = 40)	Active (*n* = 40)	*p* Value
Gender (m/f)	13/27	12/28	0.809
Age (years)	62.05 (1.55)	61.88 (1.36)	0.593
BMI (kg/m^2^)	26.48 (2.03)	27.23 (2.49)	0.145
SBP (mmHg)	129.62 (10.0)	131.55 (7.41)	0.329
DBP (mmHg)	83.28 (7.12)	83.45 (5.51)	0.902
TC (mg/dL)	203.93 (42.15)	202.35 (39.74)	0.864
LDL-C (mg/dL	134.85 (38.40)	130.33 (35.22)	0.584
HDL-C (mg/dL)	54.40 (11.02)	55.48 (14.13)	0.705
TG (mg/dL)	115.18 (56.30)	120.25 (70.31)	0.722
Glucose (mg/dL)	85.08 (16.58)	83.05 (13.46)	0.550
Insulin (mU/L)	10.70 (8.23)	8.60 (4.09)	0.153
HOMA-IR	2.27 (1.90)	1.80 (0.95)	0.162
Uricacid (mg/dL)	4.93 (1.12)	4.79 (1.48)	0.616
MDA (pmol/mL)	91.30 (52.31)	85.44 (38.59)	0.570
8-isoprostanes (pg/mL)	160.44 (162.18)	147.60 (151.79)	0.716
MMSE	28.67 (0.57)	28.64 (0.51)	0.831
TMT-B (s)	54.64 (21.30)	56.97 (25.37)	0.794
TMT-A (s)	24.20 (7.37)	25.17 (10.53)	0.814
TMT-B minus TMT-A (s)	30.44 (17.96)	31.80 (22.32)	0.799
MOCA	25.18 (1.82)	25.50 (1.83)	0.428
GDS	1.93 (1.19)	1.83 (1.28)	0.718

BMI: body mass index; SBP: systolic blood pressure; DBP: diastolic blood pressure; TC: total cholesterol; LDL-C: low density lipoprotein cholesterol; HDL-C: high density lipoprotein cholesterol; TG: triglycerides; HOMA-IR: homeostasis model assessment–insulin resistance; MMSE: mini mental state examination; TMT: trail making test; MOCA: Montreal cognitive assessment; GDS: geriatric depression scale. Data are presented as means (SD).

**Table 2:** Changes of neuropsychological tests score during the study period vs. baseline (ITT population).

	Placebo (*n* = 40) Week 8	Active (*n* = 38) Week 8	*p* Value
TMT B (s)	3.46 (13.08)	−17.63 (18.93)	<0.0001
TMT A (s)	−0.37 (5.31)	−6.86 (10.00)	<0.0001
TMT B-A (s)	3.84 (11.65)	−10.46 (16.62)	<0.0001
VFT letter (n)	1.07 (4.80)	5.28 (6.25)	0.0009
VFT category (n)	0.46 (1.79)	0.74 (1.67)	0.4915
AVL (n)	−0.13 (49.02)	−4.87 (43.09)	0.750
Delayed AVL (n)	4.60 (11.42)	4.38 (12.84)	0.591
MOCA	0.55 (2.59)	1.13 (2.17)	0.150
MMSE	0.03 (0.16)	0.03 (0.16)	0.907

TMTB: trail making test B; TMTA: trail making test A; TMT B-A: trail making test B minus trail making test A; VFT: verbal fluency test; AVL: auditory verbal learning; MOCA: Montreal cognitive assessment; MMSE: mini mental state examination.

**Table 3:** Changes of metabolic parameters during the study period vs. baseline (ITT population).

	Placebo (*n* = 40) Week 4	Active (*n* = 38) Week 4	*p* Value	Placebo (*n* = 40) Week 8	Active (*n* = 38) Week 8	*p* Value
SBP (mmHg)	0.52 (2.46)	0.13 (2.65)	0.745	0.54 (2.08)	1.18 (2.45)	0.108
DBP (mmHg)	0.42 (2.14)	−0.49 (2.72)	0.122	0.33 (2.32)	0.03 (2.72)	0.719
TC (mg/dL)	0.70 (29.50)	1.77 (28.14)	0.864	−0.18 (31.51)	3.95 (30.36)	0.563
LDL-C (mg/dL)	0.15 (29.11)	2.31 (23.13)	0.965	−1.38 (31.30)	5.03 (26.07)	0.432
HDL-C (mg/dL)	−1.15 (8.13)	0.10 (9.79)	0.349	2.13 (9.67)	2.08 (11.54)	0.819
TG (mg/dL)	3.20 (47.41)	−6.26 (49.30)	0.435	−0.13 (49.02)	−4.87 (43.09)	0.750
Glucose (mg/dL)	3.10 (10.81)	7.03 (12.16)	0.164	4.60 (11.42)	4.38 (12.84)	0.591
Insulin (mU/L)	−1.57 (5.41)	0.99 (5.42)	0.116	−2.31 (6.50)	0.70 (3.51)	0.036
HOMA-IR	−0.27 (1.19)	0.32 (1.23)	0.095	−0.41 (1.56)	0.25 (0.90)	0.080
Uric acid (mg/dL)	0.07 (0.89)	0.22 (0.76)	0.530	−0.06 (1.13)	0.01 (0.99)	0.915

SBP: systolic blood pressure; DBP: diastolic blood pressure; TC: total cholesterol; LDL-C: low density lipoprotein cholesterol; HDL-C: high density lipoprotein cholesterol; TG: triglycerides; HOMA-IR: homeostasis model assessment–insulin resistance. Data are presented as means (SD).

## Data Availability

The data presented in this study are available on request from the corresponding author. The data are not publicly available due to privacy or ethical restrictions.

## Appendix A

This appendix has been provided by the authors to give readers additional information about their work

### Appendix A.1

**Table A1:** Food supplement tablet composition.

Average Amounts of Characterizing Ingredients Quantity	Quantity	%NRVs
	(mg/dose)	
Astaxanthin	160,000	15,610
Lycopene	100,000	9756
Bacopa (Bacopa monnieri (L.) Pennel) aerial part e.s. tit. 50% bacosides	80,000	7805
Cyanocobalamin (vitamin B12)	6000	0.585
Microcrystalline cellulose	619,434	60,433
Coating agent: hydroxypropylmethylcellulose	15,819	1543
Antiagglomerating agents: crosslinked sodium carboxymethylcellulose	15,000	1463
Antiagglomerating agents: polyethylene glycol	8500	0.829
Antiagglomerating agents: magnesium vegetable stearate	8000	0.780
Antiagglomerating agents: silicon dioxide	6000	0.585
Dye: titanium dioxide	3651	0.356
Antiagglomerating agent: vegetable stearic acid	2434	0.237
Dye: oxide of iron (red)	0.150	0.015
Dry: Patent blue V	0.013	0.001
Total	1,025,000	100,000

**Table A2:** Placebo tablet composition.

Average Amounts of Characterizing Ingredients Quantity	Quantity	% NRVs
	(mg/dose)	
Microcrystalline cellulose	994,434	97,018
Coating agent: hydroxypropylmethylcellulose	15,819	1.543
Antiagglomerating agents: magnesium vegetable stearate	5.000	0.488
Dye: titanium dioxide	3.651	0.356
Antiagglomerating agents: silicon dioxide	3.000	0.293
Antiagglomerating agent: vegetable stearic acid	2.434	0.237
Antiagglomerating agents: polyethylene glycol	0.500	0.049
Dye: oxide of iron (red)	0.150	0.015
Dry: Patent blue V	0.013	0.001
Total	1,025,000	100,000

### Appendix A.2

Cognitive Performances Assessment

Cognitive performances have been evaluated by TMT, VFT, MoCA, MMSE, and AVLT.

- TMT is a frequently used neuropsychological test because of its sensitivity to brain damage. It explores visual–conceptual and visual–motor tracking. TMT is administered in two parts. Part A is a visual-scanning, timed task where participants are asked to connect with lines 25 circles numbered from 1 to 25, as quickly as possible. The test is terminated after 5 min even if not completed. In Part B participants are asked to connect circles containing numbers (from 1 to 13) or letters (from A to L) in an alternate numeric/alphabetical order. The test is terminated in every case after 10 min even if not completed. The TMT B minus TMT A score, calculated as the difference between TMT B and TMT A times, is considered a measure of cognitive flexibility, relatively independent of manual dexterity.
- VFT is a short test of verbal functioning. Participants are given 1 min to produce as many unique words as possible within a semantic category (category fluency) or starting with a given letter (letter fluency). The participant’s score in each task is the number of unique correct words.
- MoCA evaluates a broader array of cognitive domains (e.g., attention/executive functioning, visuospatial abilities, and language) and it has been demonstrated to be able to detect cognitive impairment with scores ranging from 0 to 30.
- MMSE is a widely used screening tool for cognitive impairment and covers five areas of cognitive function including orientation, attention, calculus, recall, and language with scores ranging from 0 to 30.
- AVLT is a neuropsychological assessment designed to evaluate the nature and severity of memory dysfunction, and to track changes in memory function. The examiner reads aloud a list of 15 words at the rate of one per second. The participant is then asked to repeat all words from the list that she/he can remember. This procedure is carried out a total of five times. After a 15-min delay, the participant is again asked to recall as many words as possible from the first list. The participant is then requested to read a list of words, and asked to indicate whether each word was from the first list. The score for each trial is the number of words correctly recollected.
